# Mindfulness Is Associated with Lower Depression, Anxiety, and Post-Traumatic Stress Disorder Symptoms and Higher Quality of Life in Patients with an Implantable Cardioverter–Defibrillator—A Cross-Sectional Study

**DOI:** 10.3390/healthcare12111118

**Published:** 2024-05-30

**Authors:** Marc Dörner, Roland von Känel, Noelle König, Aju P. Pazhenkottil, Rahel Altwegg, Ladina Nager, Veronica Attanasio, Lisa Guth, Sina Zirngast, Anna Menzi, Claudia Zuccarella-Hackl, Mary Princip

**Affiliations:** 1 Department of Consultation-Liaison-Psychiatry and Psychosomatic Medicine, University Hospital Zurich, University of Zurich, 8091 Zurich, Switzerland; roland.vonkaenel@usz.ch (R.v.K.); noelle.koenig@gmail.com (N.K.); aju.pazhenkottil@usz.ch (A.P.P.); rahel.altwegg@usz.ch (R.A.); ladina.nager@usz.ch (L.N.); veronica.attanasio@usz.ch (V.A.); lisa.guth@usz.ch (L.G.); sina.zirngast@usz.ch (S.Z.); menzi.anna@gmail.com (A.M.); claudia.hackl-zuccarella@usz.ch (C.Z.-H.); mary.princip@usz.ch (M.P.); 2 German Center for Neurodegenerative Diseases (DZNE) within the Helmholtz Association, 39120 Magdeburg, Germany

**Keywords:** implantable cardioverter–defibrillator, depression, anxiety, post-traumatic stress disorder, mental health, mindfulness, quality of life

## Abstract

Several studies suggested the efficacy of dispositional mindfulness and mindfulness-based interventions in reducing anxiety and depression in cardiovascular diseases. However, data on the impact of mindfulness on the psychological well-being of patients with an implantable cardioverter–defibrillator (ICD) are scarce. In this study, 422 patients with an ICD were prospectively recruited. Logistic regression was applied to determine associations between dispositional mindfulness (Freiburg Mindfulness Inventory), depression (Patient Health Questionnaire-8), anxiety (Generalized Anxiety Disorder-7 scale), and post-traumatic stress disorder (PTSD) symptoms (Post-Traumatic Stress Diagnostic Scale), adjusting for age, sex, educational status, number of ICD shocks after ICD implantation, and physical activity. The PROCESS regression path analysis modelling tool was used to identify indirect mediating effects of dispositional mindfulness on depression, anxiety, and PTSD symptoms and quality of life (QoL; EuroQol group 5-dimension questionnaire). Participants presented high baseline QoL (mean 1.06 to 1.72) and medium–high mindfulness scores (mean 40.85 points). Higher mindfulness scores were associated with lower levels of anxiety (OR 0.90, 95% CI 0.86 to 0.95, 0.001), depression (OR 0.93, 95% CI 0.88 to 0.98, *p* = 0.006), and PTSD symptoms (OR 0.94, 95% CI 0.89 to 0.98, *p* = 0.011). Furthermore, greater mindfulness partially mediated the relationship between anxiety (indirect effect 0.10, 95% CI 0.02 to 0.21), depression (indirect effect 0.08, 95% CI 0.01 to 0.17), or PTSD (indirect effect 0.04, 95% CI 0.01 to 0.17) as independent variables and the QoL as the dependent variable. This study suggests that greater dispositional mindfulness is associated with less anxiety, depression, and PTSD symptoms. Mindfulness might also increase the QoL in ICD patients by mitigating the impact of those with psychological distress.

## 1. Introduction

An implantable cardioverter–defibrillator (ICD) treats and prevents life-threatening conditions of the cardiovascular system, such as ventricular arrhythmias, cardiac arrest, or sudden cardiac death, by anti-tachycardia pacing or shocks. On the other hand, previous studies suggested worse emotional functioning and a reduced quality of life (QoL) after ICD implantation and ICD therapy [1]. Furthermore, 12% to 22% of ICD patients show signs of post-traumatic stress disorder (PTSD), depression, or anxiety [2,3], and these psychiatric symptoms and disorders are associated with increased mortality rates in those patients [4].

Therefore, interventions or skills to mitigate the potential devastating psychological consequences of ICD implantation or therapy and to improve ICD patients’ QoL are needed. For example, mindfulness might be such an approach. Central to Eastern philosophies for centuries, the concept of mindfulness originated in earliest Buddhist documents. This concept of mindfulness is characterised by non-judgmental and present-moment awareness, including continuous awareness of physical sensations and affective as well as mental states. Mindfulness implies a form of naturalistic observation with the following assumptions: (a) human beings are usually not aware of their present experience, often operating in an absentminded mode; (b) developing the ability of mindfulness is gradual and progressive and can be learned through regular practice; (c) a present-moment awareness will lead to a more vital and richer sense of life; (d) and finally, this more precise perception of own mental responses to internal and external stimuli will lead to greater self-control and thus more effective conduct in the world. Consequently, this should result in enhanced emotional processing and coping skills with regard to chronic diseases and stress, increased self-control, and a more nuanced interpretation of the meaning of well-being, i.e., in the presence of stress and suffering, a rich and full life is still possible [5]. During the last decades, mindfulness-based interventions have also been successfully implemented in western medical settings. Indeed, mindfulness practice has been demonstrated to decrease depression, anxiety, sleep disturbances, and several physical symptoms in healthy populations and in patients with cancer, chronic pain, and cardiovascular diseases [5,6,7,8].

For example, in cardiovascular diseases, anxiety might play a significant role on morbidity and mortality due to an increased activity of the sympathetic nervous system and an increased incidence of severe arrhythmias. On the other hand, mindfulness has been shown to reduce the activity of the sympathetic nervous system, arrhythmias, and anxiety in patients with coronary heart disease or heart failure [9]. 

However, data on the influence of mindfulness on the psychological well-being of ICD patients are scarce. One study including adult ICD patients highlighted a significant association between higher dispositional mindfulness, lower anxiety scores, and no history of depression in univariate analyses, which diminished to non-significant effects after adjusting for other covariates [8]. In a second study, the same study group observed a non-significant trend towards decreased anxiety after mindfulness training compared to the control group [9]. On the other hand, another study in adolescents with an ICD found a significant decrease in anxiety with a mindfulness-based intervention [10]. Additionally, a recent study did not indicate any impact of a mindfulness-based intervention on anxiety, depression, or QoL in adult patients with an ICD [11]. The relationship between mindfulness and PTSD symptoms has not previously been investigated in ICD patients.

Due to these inconsistent findings with relatively small sample sizes and given that ICD patients often suffer from PTSD, depression, or anxiety and a reduced QoL, which are associated with an increased mortality rate, replication studies are warranted. Moreover, ICD patients tend to be more attentive to their physical symptoms (such as chest pain, shortness of breath, palpitations), which could point to an exacerbation of their condition, and thus, they learn to be more aware of physical sensations and what unveils in the presence [9]. Hence, our interests were two-fold. First, we aimed to describe the baseline levels of dispositional mindfulness, psychological variables, and QoL in an ICD population. Second, we aimed to explore the associations between mindfulness, anxiety, depression, and QoL. In contrast to the already existing studies [8,9,10,11], we included a larger sample size, further analysed possible links between mindfulness and PTSD as well as other biomedical variables, such as ICD shocks, and performed a mediational analysis as a novel approach.

## 2. Materials and Methods

### 2.1. Setting and Sample

**Figure 1:**
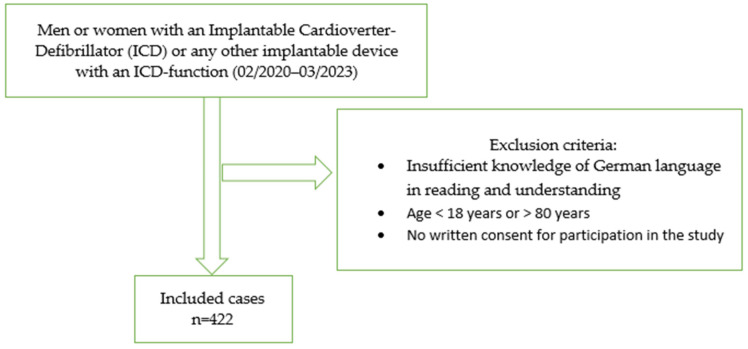
Process of selecting the study sample.

We prospectively included in this national single-centre cross-sectional study a sample of 422 participants with an implantable device with ICD function. Participants were recruited at their half-yearly routine check-up at the Cardiac Arrhythmia Division (Department of Cardiology) at the University Hospital Zurich between February 2020 and March 2023. Exclusion criteria were age < 18 years or >80 years, insufficient knowledge of the German language, and no written consent for participation in this study (see Figure 1 for procedure of study sample selection). Self-report questionnaires regarding psychometric variables, sociodemographics, medical-related variables, and ICD concerns were filled out by the study participants. All participants provided written informed consent. This study was approved by the Cantonal Ethics Committee of Zurich (no. 2019-01948, 12/2019).

### 2.2. Measurements

To detect symptoms of depression, we utilized the Patient Health Questionnaire-8 (PHQ-8). Items of the PHQ-8 are scored from 0 (absent) to 3 points (severe), consisting of the diagnostic criteria for major depressive disorder (MDD). A cut-off score of 10 or higher indicates MDD [12]. In contrast to the PHQ-9, the PHQ-8 does not inquire about suicidal ideation.

We used the Generalized Anxiety Disorder-7 (GAD-7) scale for the identification of anxiety symptoms. The seven items of the GAD-7 range from 0 (not at all) to 3 (nearly every day), with a cut-off score of 10 or higher indicating generalized anxiety disorder (GAD) [13].

For the identification of PTSD symptoms, we applied the Post-Traumatic Stress Diagnostic Scale (PDS). It consists of 17 items including the cardinal symptoms of PTSD. Respondents with a cut-off score of 14 or higher are suggested to exhibit PTSD [14]. 

Individual differences in dispositional mindfulness were measured with the short form of the German version of the Freiburg Mindfulness Inventory (FMI, 14 items). Higher scores indicate higher levels of mindfulness (range 14 to 56 points) [15]. 

The health-related QoL was measured using the German version of the EuroQol group 5-dimension questionnaire (EQ-5D-5L). It considers five dimensions of health including mobility, self-care, usual activities, pain or discomfort, as well as anxiety or depression [16]. Each item is scored from 1 (no problems) to 5 (extreme problems). Lower scores on each dimension indicate a higher QoL. Of note, the PHQ-8, the GAD-7, and the dimension “anxiety or depression” of the EQ-5D-5L did not show a significant correlation with each other.

### 2.3. Statistical Procedures

For statistical analysis, we used IBM SSPSS Statistics for Windows, Version 29 (Armonk, NY: IBM Corp). We calculated mean and median scores, standard deviation, and relative and absolute distributions to describe patient characteristics. An unpaired t-test or a one-way ANOVA was used to compare normally distributed continuous variables and a chi-square test to compare categorical variables. Binary logistic regression was performed to investigate associations between depression (PHQ-8 score ≥ 10), anxiety (GAD-7 score ≥ 10), or PTSD (PDS score ≥ 14) as the dependent variable, mindfulness as the independent variable, and age, sex, educational status, the number of ICD shocks after ICD implantation, and regular physical activity “that makes you sweat” (yes or no) as covariates. Collinearity statistics were applied, but did not indicate any issues of multicollinearity (variance inflation factor < 2.5 and tolerance > 0.4, [17]). Moreover, the PROCESS regression path analysis modelling tool for SPSS was used to explore indirect mediating effects of mindfulness between depression, anxiety, or PTSD as the independent variable and the QoL as the dependent variable. Significance level (two-sided *p*-value) was set at *p* < 0.05.

## 3. Results

### 3.1. Sample

Table 1 depicts sociodemographic and clinical characteristics of the study sample and the amount of missing data for each variable. The majority of participants were men, who were significantly older, had significantly more often experienced myocardial infarction, and demonstrated significantly higher levels of mindfulness compared to women. On the other hand, women had significantly higher levels of PTSD symptoms and tended to have a lower health-related QoL than men.

Participants with a higher educational status had significantly higher mindfulness levels. Mindfulness differences between participants who experienced at least one ICD shock in the past (*n* = 126, 30.65%) compared to those who never had any ICD shock (*n* = 285, 69.34%) were not significant (Table 2). Those participants, who reported lower QoL regarding usual activities, pain or discomfort, and anxiety or depression, had significantly lower mindfulness levels. However, mindfulness levels were not significantly different between patients with high or low QoL-related mobility and self-care (see Supplementary Table S1).

### 3.2. Associations between Mindfulness, Anxiety, Depression, and PTSD

Table 3 illustrates the results of the significant binary logistic regression models (*p* < 0.001), with an explained variance (Nagelkerke R^2^) ranging from 10% to 21%. Older participants indicated significantly higher levels of anxiety and PTSD. Regular physical activity was associated with significantly lower anxiety scores. An increasing number of ICD shocks after ICD implantation predicted significantly higher levels of anxiety, depression, and PTSD. Higher scores of mindfulness showed significantly lower odds of having anxiety, depression, or PTSD.

### 3.3. Mediation Effects of Mindfulness on Depression, Anxiety, PTSD, and Quality of Life

**Figure 2:**
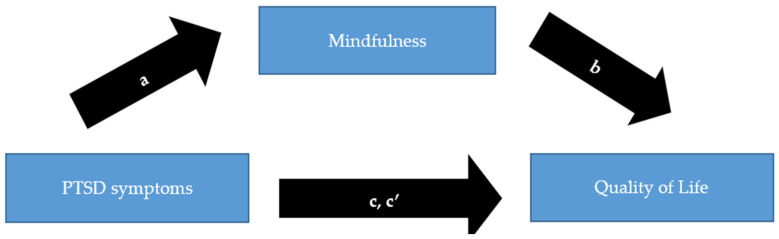
Mediation model testing indirect effects of mindfulness on post-traumatic stress disorder (PTSD) symptoms and quality of life (QoL). The Post-Traumatic Stress Diagnostic Scale (PDS) score was the independent, the QoL score the dependent variable, and the mindfulness score the mediator. Covariates were age, sex, educational status, physical activity, and the number of implantable cardioverter–defibrillator (ICD) shocks after ICD implantation. a: effect of PTSD symptoms on mindfulness (−3.28, 95% confidence interval (CI) −6.11 to −0.44, *p* = 0.02). b: effect of mindfulness on QoL (−0.06, 95% CI −0.10 to −0.02, *p* < 0.001). c’: direct effect of PTSD symptoms on QoL (3.17, 95% CI 2.20–4.15, *p* < 0.001). c: total effect of PTSD symptoms on QoL (3.36, 95% CI 2.38–4.35, *p* < 0.001).

Figure 2 displays the mediation analysis between PTSD as the independent, QoL as the dependent variable, and mindfulness as the mediator. This model indicated significant effect sizes of mindfulness as the partial mediator mitigating the impact of PTSD symptoms on the QoL (indirect effect 0.04, 95% confidence interval (CI) 0.01 to 0.17). The same model also revealed significant partial mediating effects of mindfulness for depression (indirect effect 0.08, 95% CI 0.01 to 0.17) and anxiety (indirect effect 0.10, 95% CI 0.02 to 0.21) as independent variables.

## 4. Discussion

In this naturalistic study, we analysed the level of dispositional mindfulness, QoL, and psychological as well as biomedical variables in an ICD population. Furthermore, we explored associations between mindfulness, anxiety, depression, and PTSD. Finally, we tested indirect mediating effects of mindfulness on these psychiatric conditions and QoL.

ICD patients demonstrated medium–high levels of dispositional mindfulness and an overall high QoL, which is in line with previous studies [11,18,19]. This finding, together with a lower prevalence of depression and anxiety and similar levels of PTSD compared to results of a recent meta-analysis in ICD patients [2], indicates that having an ICD does not necessarily lead to a reduced QoL. Still, several studies suggested that ICD therapy, i.e., received shocks, are a main contributing factor for reduced QoL and increased anxiety and depression in patients with an ICD [1,20,21]. However, in particular, this association seems to be relevant in the early period after ICD implantation [22], while our study comprised both early and late post-ICD implantation periods. A history of ICD shocks predicted anxiety, depression, and PTSD in our study sample, indicating that there might be a subgroup of ICD patients who are particularly vulnerable to the psychological consequences of ICD implantation and therapy. 

Interestingly, there was no difference of mindfulness between those patients who experienced at least one ICD shock in the past and those who did not. In theory, a potential life-threatening event triggering an ICD shock might lead to an attitude of increased mindfulness in patients’ everyday life, who become more sensitive to physical symptoms and sensations related to their condition. Studies with other patient cohorts reported an increased appreciation of the value of life with an elevated focus on the present moment and interpersonal growth [23]. Our finding that participants with a higher educational status seem to be more mindful than those with a lower education confirms results from other studies. Those implied that especially people with a highly educated and high socioeconomic status are executing mindfulness-based interventions [24]. 

Overall, greater mindfulness was associated with significantly lower levels of depression, anxiety, and PTSD even after adjusting for several covariates. In agreement with other studies, this observation supports the existing evidence that mindfulness plays a pivotal role in psychological well-being as a potential variable of resistance, empowering patients to withstand the mental sequelae of an ICD implantation [25,26,27,28,29]. Moreover, a lower health-related QoL with regard to usual activities, pain or discomfort, and anxiety or depression was indicative of significantly lower mindfulness levels. Yet, our mediation models predicted significant indirect effects of mindfulness, mitigating the impact of the psychological factors (anxiety, depression, and PTSD) associated with an ICD and thus improving the QoL in those patients. This means that mindfulness could also be a variable of resilience, giving patients the ability to recover from the disturbances caused by an ICD. These potential positive effects of mindfulness on the psychological well-being of this patient group might be explained by a more vital and richer experience of life and an increased self-control via a higher perception of their own mental responses to inner stimuli and stimuli of the external world. Then, ICD patients’ abilities to cope with stress and emotional processes might enhance [5]. On a physiological level, a reduction of the sympathetic nervous system activity with a decreased number of potential arrhythmias could further increase the QoL in patients with an ICD [9].

Compared to the other studies that investigated the impact of mindfulness in ICD patients [8,9,10,11], the current study consisting of a much larger study sample allows a more nuanced interpretation of the results. This study also added new knowledge of mindfulness in ICD patients by examining further variables of interest. However, there are some limitations to this study. First, this study was limited to a single centre and a highly selected sample, which restricts generalisability. Second, the exclusion of patients with insufficient German language skills could be a potential bias. Of course, there might be other important aspects future studies should consider in their analyses, such as different cultural backgrounds or nationalities, that might have an impact on the efficacy of mindfulness. Third, longitudinal data will be required to evaluate real causal effects of mindfulness on the psychological well-being of ICD patients. Path analyses can yield only a proxy estimate of causality. Fourth, clinically relevant symptoms derived from cut-off scores cannot replace a psychiatric diagnosis based on a clinical interview [30]. Fifth, we had no information whether psychiatric symptoms were already present before ICD implantation. There were a few missing values for some examined variables. However, given the relatively low number of missing values, ranging from 1.7% to 10.2% depending on the examined variable, we do not assume that this leads to biased results. Finally, mindfulness was self-reported and adequate definitions of mindfulness are often not provided and may differ across studies [31].

## 5. Conclusions

This study suggests that greater dispositional mindfulness is associated with less anxiety, depression, and PTSD symptoms in patients with an ICD. Furthermore, mindfulness might lead to an increased QoL by mediating the effects of psychological sufferings. Future studies with adequate sample sizes should strive to test the effects of mindfulness-based interventions in ICD populations, which may shed further light on the mechanisms and potential beneficial effects of mindfulness on mental health outcomes. The effects of mindfulness on other psychological sufferings that are associated with ICD patients might be further explored, preferably in large multi-centre and randomized clinical trials. In particular, prospective studies could expand our novel research of the impact of mindfulness on PTSD symptoms, which are often found in ICD patients. 

## Figures and Tables

**Table 1:** Description of the study sample.

	Overall (*n* = 422)	Women (*n* = 103)	Men (*n* = 319)	*p*-Value (*p* < 0.05)
Age (y), mean (SD)	57.77 (13.96)	52.13 (14.88)	59.58 (13.16)	<0.001 *
Educational status	*n* = 415 (%)	*n* = 101	*n* = 314	<0.001 *
Lower than completed apprenticeship or equivalent	17 (4.09)	12 (11.88)	5 (1.59)	
Completed apprenticeship or equivalent	235 (56.62)	54 (53.46)	181 (57.64)	
High-school diploma or equivalent	62 (14.93)	17 (16.83)	45 (14.33)	
University degree	101 (24.33)	18 (17.82)	83 (26.43)	
	*n* = 409 (%)	*n* = 99	*n* = 310	
Past myocardial infarction	155 (37.89)	14 (14.14)	139 (44.83)	<0.001 *
	*n* = 379	*n* = 86	*n* = 293	
Number of ICD shocks in the past, mean (SD)	1.20 (5.35)	1.28 (3.78)	1.18 (5.80)	0.782
	*n* = 416 (%)	*n* = 103	*n* = 313	
Regular physical activity	237 (56.97)	59 (57.28)	178 (56.86)	0.942
	*n* = 408 (%)	*n* = 102	*n* = 306	
Anxiety (GAD-7 ≥ 10)	37 (9.06)	14 (13.72)	23 (7.51)	0.059
	*n* = 410 (%)	*n* = 98	*n* = 312	
Depression (PHQ-8 ≥ 10)	42 (10.24)	12 (12.24)	30 (9.61)	0.454
	*n* = 415 (%)	*n* = 102	*n* = 313	
PTSD (PDS ≥ 14)	54 (13.01)	21 (20.58)	33 (10.54)	0.009 *
QoL, mean (SD)	*n* = 422	*n* = 103	*n* = 319	
Mobility	1.55 (0.90)	1.46 (0.82)	1.57 (0.92)	0.259
Self-care	1.06 (0.36)	1.02 (0.13)	1.08 (0.40)	0.027 *
Usual activities	1.53 (0.84)	1.69 (0.88)	1.48 (0.82)	0.028 *
Pain or discomfort	1.72 (0.87)	1.75 (0.87)	1.72 (0.87)	0.750
Anxiety or depression	1.48 (0.76)	1.64 (0.83)	1.43 (0.73)	0.015 *
Mindfulness, mean (SD)	40.85 (7.30)	38.67 (7.35)	41.53 (7.16)	<0.001 *

Note: Mean values are reported for age, QoL, and mindfulness scores. The other variables are presented as absolute numbers and relative (%) values. Information regarding educational status, past myocardial infarction, number of ICD shocks in the past, regular physical activity, anxiety, depression, and PTSD were available for most but not all participants. n: number. y: years. SD: standard deviation. ICD: implantable cardioverter–defibrillator. GAD-7: Generalized Anxiety Disorder-7. PHQ-8: Patient Health Questionnaire-8. PDS: Post-Traumatic Stress Diagnostic Scale. QoL: quality of life. * Significant *p*-values are marked bold.

**Table 2:** Mindfulness scores compared to other clinical variables.

	Mindfulness, Mean (SD)	*p*-Value (*p* < 0.05)
Educational status	*n* = 414	0.032 *
Lower than completed apprenticeship or equivalent	36.65 (7.47)	
Completed apprenticeship or equivalent	40.63 (7.65)	
High-school diploma or equivalent	41.26 (6.89)	
University degree	42.03 (6.24)	
	*n* = 404	0.461
Past myocardial infarction, yes	41.27 (7.58)	
no	40.72 (7.07)	
	*n* = 411	0.910
ICD shock in the past, yes	40.87	
no	40.96	
	*n* = 411	0.271
Regular physical activity, yes	41.18 (7.07)	
no	40.38 (7.58)	

Note: Information regarding educational status, past myocardial infarction, ICD shock in the past, and regular physical activity were available for most but not all participants. * Significant *p*-values are marked bold.

**Table 3:** Binary logistic regression for GAD (GAD-7 ≥ 10), MDD (PHQ-8 ≥ 10), and PTSD (PDS ≥ 14) in patients with an ICD.

	GAD		MDD		PTSD	
Variables	OR (95% CI)	*p*-Value (*p* < 0.05)	OR (95% CI)	*p*-Value (*p* < 0.05)	OR (95% CI)	*p*-Value (*p* < 0.05)
Male sex	0.86 (0.34 to 2.15)	0.756	0.96 (0.40 to 2.29)	0.931	1.54 (0.74 to 3.22)	0.245
Age	0.95 (0.92 to 0.98)	0.004 *	0.98 (0.96 to 1.01)	0.376	0.96 (0.94 to 0.99)	0.009 *
Higher educational status	0.84 (0.52 to 1.34)	0.471	1.06 (0.70 to 1.61)	0.755	0.85 (0.57 to 1.25)	0.419
ICD shock number	1.06 (1.01 to 1.10)	0.010 *	1.05 (1.00 to 1.10)	0.019 *	1.07 (1.01 to 1.13)	0.015 *
Physical activity	0.41 (0.17 to 0.96)	0.041 *	0.55 (0.26 to 1.18)	0.131	0.67 (0.33 to 1.36)	0.273
Mindfulness	0.90 (0.86 to 0.95)	<0.001 *	0.93 (0.88 to 0.98)	0.006 *	0.94 (0.89 to 0.98)	0.011 *
Nagelkerke R^2^	0.21		0.10		0.16	

Note: OR: odds ratio. CI: confidence interval. MDD: major depressive disorder. * Significant *p*-values are marked bold.

## Data Availability

The data presented in this study are available on request from the corresponding author.

## Supplementary Materials

The following supporting information can be downloaded at: https://www.mdpi.com/article/10.3390/healthcare12111118/s1, Table S1: Mindfulness scores and quality of life.
