# Supplementary material for: Mindfulness Is Associated with Lower Depression, Anxiety, and Post-Traumatic Stress Disorder Symptoms and Higher Quality of Life in Patients with an Implantable Cardioverter–Defibrillator—A Cross-Sectional Study
Source: Healthcare (Basel). 2024 May 30;12(11):1118. doi: 10.3390/healthcare12111118 (PMC11172204; doi:10.3390/healthcare12111118)
Supplement: Supplementary file 1 [file healthcare-12-01118-s001.zip › healthcare-3027386-supplementary.pdf]

Supplementary Table S1. Mindfulness scores and quality of life.

	Mindfulness, mean (SD)	p-value (p<0.05)
Quality of Life, mean (SD)	n=414	
Mobility		0.145
No problems	41.48 (6.90)	
Slight problems	39.66 (6.21)	
Moderate problems	39.50 (9.78)	
Great Problems	40.32 (8.90)	
Extreme problems	37.00 (5.09)	
Self-care		0.192
No problems	41.02 (7.20)	
Slight problems	37.62 (7.00)	
Moderate problems	36.67 (9.45)	
Great Problems	/	
Extreme problems	36.00 (1.41)	
Usual Activities		**0.013***
No problems	41.70 (6.72)	
Slight problems	39.18 (7.60)	
Moderate problems	40.32 (7.92)	
Great Problems	37.17 (10.06)	
Extreme problems	37.00 (2.00)	
Pain or discomfort		**0.010***
No problems	41.63 (7.28)	
Slight problems	40.92 (6.49)	
Moderate problems	37.47 (7.27)	
Great Problems	40.06 (9.27)	
Extreme problems	37.67 (10.06)	
Anxiety or depression		**<0.001***
No problems	42.38 (6.65)	
Slight problems	39.31 (7.74)	
Moderate problems	36.15 (5.20)	
Great Problems	33.18 (7.44)	
Extreme problems	32.50 (9.19)	

Note: n: number. SD: standard deviation. *Significant p-values are marked bold.
